# Field study on the severity of photovoltaic potential induced degradation

**DOI:** 10.1038/s41598-022-26310-y

**Published:** 2022-12-21

**Authors:** Ghadeer Badran, Mahmoud Dhimish

**Affiliations:** School of Physics, Engineering and Technology, University of York, York, YO10 5DD UK

**Keywords:** Energy science and technology, Renewable energy, Solar energy, Photovoltaics, Solar cells

## Abstract

Photovoltaic (PV) systems can be affected by different types of defects, faults, and mismatching conditions. A severe problem in PV systems has arisen in the last couple of years, known as potential-induced degradation (PID). During the early installation stage of the PV system, the PID may not be noticed because it appears over time (months or years). As time passes, it becomes more apparent since the output power may drop dramatically. We studied PV modules over the course of three years that were affected by PID. An electroluminescent and thermal imaging technique helped discover the PID. PID appeared in PV modules after being in different fields for 4–8 months, resulting in a 27–39% drop in power. An anti-PID box was fitted during the second year of the PV operation to recover the PID. Accordingly, it has stabilized the power degradation, but it could not restore the performance of the affected PID modules as compared with healthy/non-PID modules.

## Introduction

Photovoltaic (PV) system reliability and durability have attracted considerable attention in recent years from the PV industry as well as investors, as PV defects and faults have become an essential factor that has been scientifically proven to reduce power generation from PV assets and cause complications. Previous research on PV fault detection and classification has greatly improved our understanding of PV partial shading conditions^1,2^, faulty power converters/inverters^3^, and dead-state battery storage^4^. Although we have gained some insight into the severity of hotspots^5^, cracks^6^, and potential induced degradation (PID)^7,8^, we are still far from understanding how severe the defects are.

Researchers have made significant contributions to the analysis of hotspots and cracks in PV modules and how they contribute to the degraded performance of PV modules^5,6^. Although PID has been explained in a variety of settings, there have been occasionally details on how PID develops outdoors and in the field where there are different environmental conditions. As an example of a PID-affected PV string, what are the actual power losses that are caused by a typical PV string?

There are two different types of PIDs, both of which are attributed to the inverter or power electronic device not having a grounding connection. As a result, it creates either a biasing of + 1000 V or a biasing of −1000 V for the PV string affected. This will lead to the PV modules leaking current from the semiconducting material to the actual framing or glass of the module as a result of the PID^9^. As a consequence, it will result in degrading the modules and resulting in a significant reduction in the amount of output power produced^10^.

There has been some prior research on PV modules that were tested under a controlled environment by applying the existing PID testing procedure: humidity 85%, PV surface temperature at 65 °C, and either a bias voltage of + 1000 V or −1000 V for at least 96 h^9–11^. The test can, of course, help us understand how PID affects PV modules. As an example^12^, shows that PID can reduce module output power by more than 30%. However, there is not much information on whether PID results in the same power losses in an outdoor setting. Additionally, it is unknown whether repairing the grounding issues of the affected PID strings will increase output power and improve quality. Through electroluminescence (EL) imaging^9,12,13^, PID can induce shunting, cracks, and breakdown of solar cells.

To identify PV PID, thermal imaging is used via drone inspection^14^. A thermal review of PV systems has become increasingly popular in today's PV market because thermal testing is less expensive than EL testing, which requires that PV strings be disconnected. Even in large PV installations, PID affected PV strings are easily distinguished as they have higher surface temperatures than non-PID strings^15,16^. Temperature increases are caused most often by leakage currents and voltage bias, especially with PID negative biasing, −1000 V.

Additionally, previous experiments on PID of PV systems are usually assessed over short-term testing, 96 h, and the degradation estimation of the affected modules is simply derived by observing the theoretical curves of the PV modules under an indoor simulation procedure (experimenting with the modules under a sun simulator). Consequently, due to the lack of field studies on PID, we investigated the impact of PID on a large-scale PV system, with a nearly 3-year dataset.

PID performance is often assessed by measuring current and voltage and comparing losses between PID affected and healthy modules^17^. As shown in^18^, PID is capable of significantly lowering the open-circuit voltage while not degrading short-circuit current. The PID affected module's shunt resistance (parallel resistance) is reduced, resulting in an open-circuit voltage drop. This can also be exemplified by the cells' shunting (blackout/dark) when EL images are captured^19–21^.

The paper examines the impact of PID on PV strings. As soon as the PV system became operational, in August 2019, until July 2021, an investigation was conducted. After nearly one year in the field, thermography drone inspection identified PV strings with PID. Compared with a healthy PV string, the output power losses of the PID affected strings were analysed. In addition to EL images, the current–voltage curves of the studied PV strings were studied under different environmental conditions. A PID box was fitted to the affected PV strings to recover the PID impact, and the results were analysed based on 12-month field measurements. Therefore, the main contributions of this study are to (i) study the impact of PID on PV strings by using long-term data measurements, which were largely taken in field conditions, (ii) investigate whether an anti-PID box can improve the performance of already affected PID PV strings, and (iii) calculate the probability of PV strings losing power resulting from PID.

## Results and discussions

### PID effect on the PV performance ratio

PV installation with 1.2 MW power capacity was tested in Barcelona, Spain. In this project, we investigated how PID develops (changes over time) and how severely they can impact module performance. Therefore, it was necessary to perform a thermal inspection to map the strings affected by PID. As a result, the required data can be extracted using the power converter unit.

**Figure 1:**
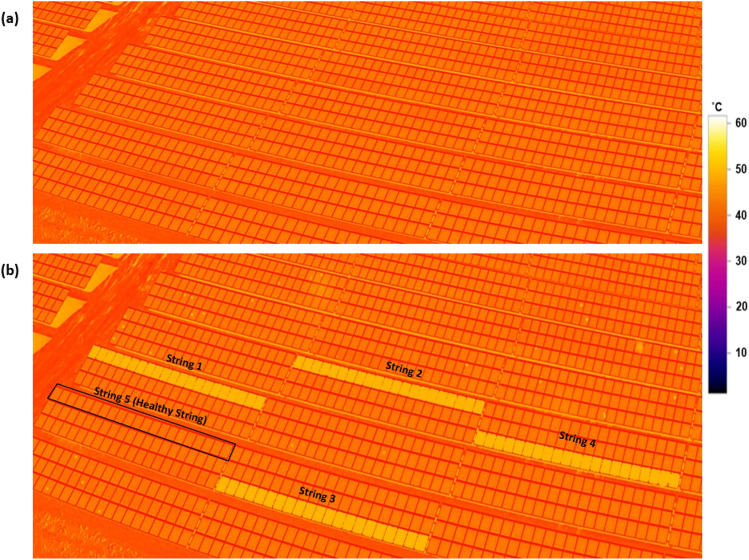
Thermography image of the tested PV strings during nigh-time. (**a)** Image taken on 01/08/2019, first day of PV operation (ambient temperature 34 °C). (**b)** Image taken on 03/08/2020, after 1-year of operation (ambient temperature 35 °C). The thermal image is taken using DJI Mavic 2 Enterprise Advanced drone with thermal sensitivity ± 2 °C.

The PV site was under construction in 2019 and was operational on 01/08/2019 when the thermal image was taken (Fig. 1a). We took these thermal images during the day when solar radiation was 712 W/m^2^, and the ambient temperature was 35 °C. No anomalies or faults were found. One year later, however, four PV strings were affected by PID, as shown in Fig. 1b. PID is characterized by an increase in PV module surface temperature to nearly 55–60 °C, compared with healthy PV strings which have a surface temperature of almost 37 °C.

The PID occurs in all PV strings due to a ground fault in the inverter, resulting in a −1000 V biasing, with each PV string containing 21 series-connected modules. In this way, the output power of the PV modules can further degrade and current can leak out. As a result, the PV modules will heat up, as shown in Fig. 1, and their output power performance will decrease over time. Note here that in the affected strings (1–4), they are all subjected to −1000 V, unlike some other reported PID effects in the literature which their PID is a resultant of + 1000 V biasing^22^. In addition, in^22^, they have also discussed that negative voltage biasing is the worst-case scenario regarding the PID effect on the PV modules, compared with either 0 V or + 1000 V biasing.

Each PV string's log of measurements was analyzed and compared with a healthy PV string (string 5, Fig. 1b). Unfortunately, the current–voltage and power-voltage curves of the individual PV strings were not measured during the thermal inspection. The healthy PV string, string 5, was carefully selected as other PV strings had already developed hotspots and other mismatching conditions. We calculated the output performance ratio (PR) for each month. The PR ratio would indicate when the PID developed in the PID-affected PV strings and how much deterioration in output power developed compared with the healthy PV strings.

**Figure 2:**
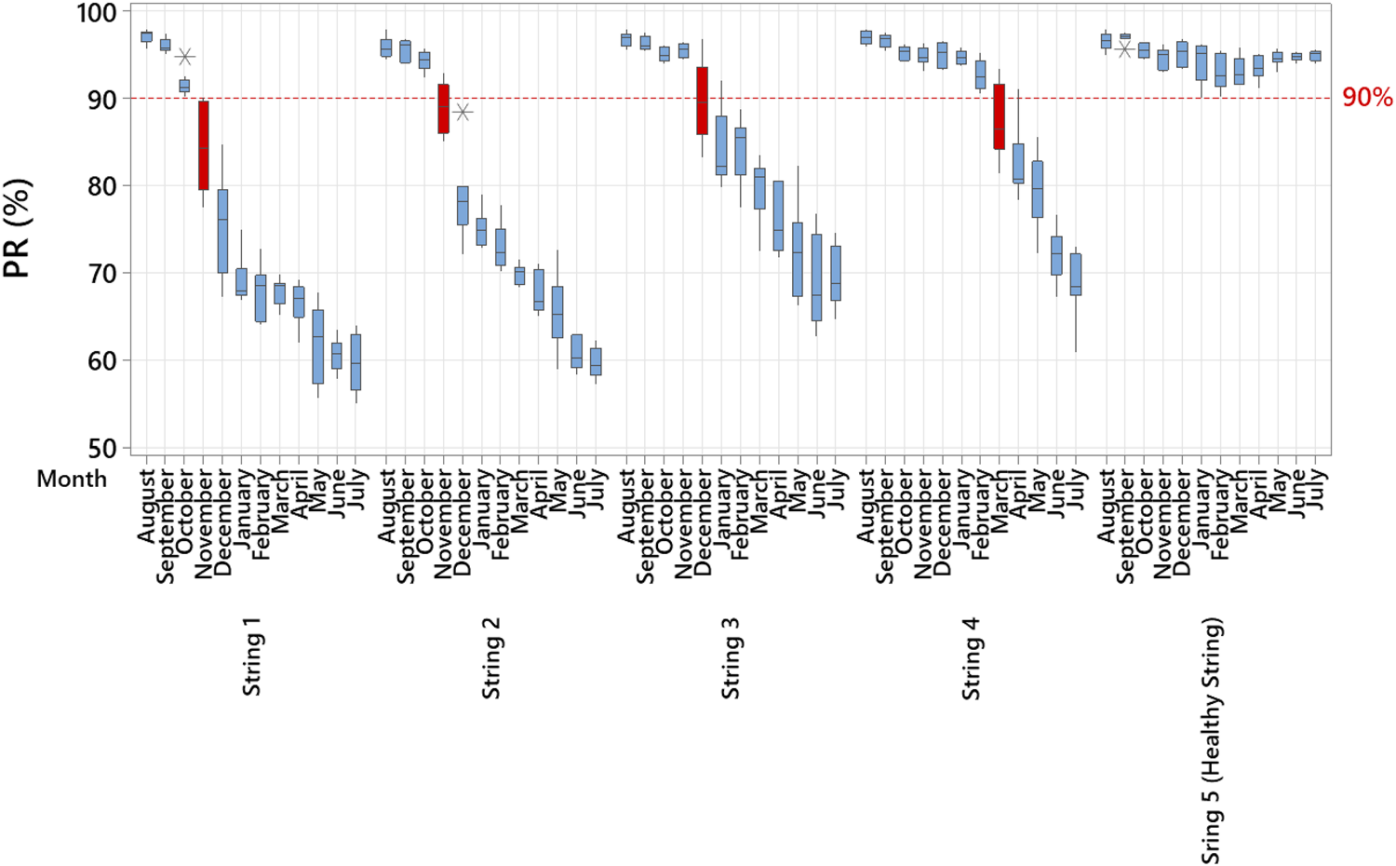
Performance ratio of the tested PV strings. The y-axis presents the PR ratio, and the baseline of 90% was selected to indicate low performance; the x-axis gives the month. This chart shows the PR ratio for the PV strings during their first year of operation and the red labelled boxplots for the individual PV strings believed to be the starting month of the PID.

The results of the monthly calculated PR ratio are presented in Fig. 2. The healthy PV string has no monthly PR ratio below 90% during the entire year of operation. However, the decline of the PR ratio of the other PV strings has been seen at different times. For example, in PV string 1, the monthly PR drop started in November (4-months after field operation); the same observation is valid for PV string 2. The third PV string has a slight decline in the PR starting from December; this suggests that PID has occurred after 5-months of field operation. In contrast, PV string 4 has no significant drop in the PR ratio during the first 7-months of field operation. However, a fast decline in its performance is evident in March, due to PID.

These results demonstrate two critical indicators on the impact of PID:

1. PID can have an instantaneous reduction in the output power of the PV strings. For example, in PV string 2, after 4-months of field operation, the PR has dropped from nearly 90% in November to 77% in December.
2. PID develops in the PV strings at different periods. This implies that the early thermal inspection (day one) is insufficient to detect PID abnormalities.

It is also clear from Fig. 2 that PIDs can accelerate during relatively hot months, May through July. Prior to our examination, there was inadequate information on whether the PID effect could still have an effect on PV modules during these months due to the lower differences between PV surface and ambient temperature. The opposite has been proven by our experiment. In addition, Table 1 shows the PR ratio difference in the first month compared with last month. The PR difference ranges from 27.9 to 39.9% for the PID-affected PV strings, while the PR difference for the healthy PV strings is only 0.3%. These results are consistent with those obtained by laboratory testing^9,12^.

**Table 1:** Comparative analysis of the PV strings PR.

PV String	PR (first month) (%)	PR (after 12-months) (%)	Difference (%)
1	96.5	56.6	39.9
2	94.8	58.3	36.5
3	96.1	66.9	29.2
4	96.3	68.4	27.9
5 (Healthy string)	95.7	94.4	0.3

### PID examination using electroluminescent imaging

**Figure 3:**
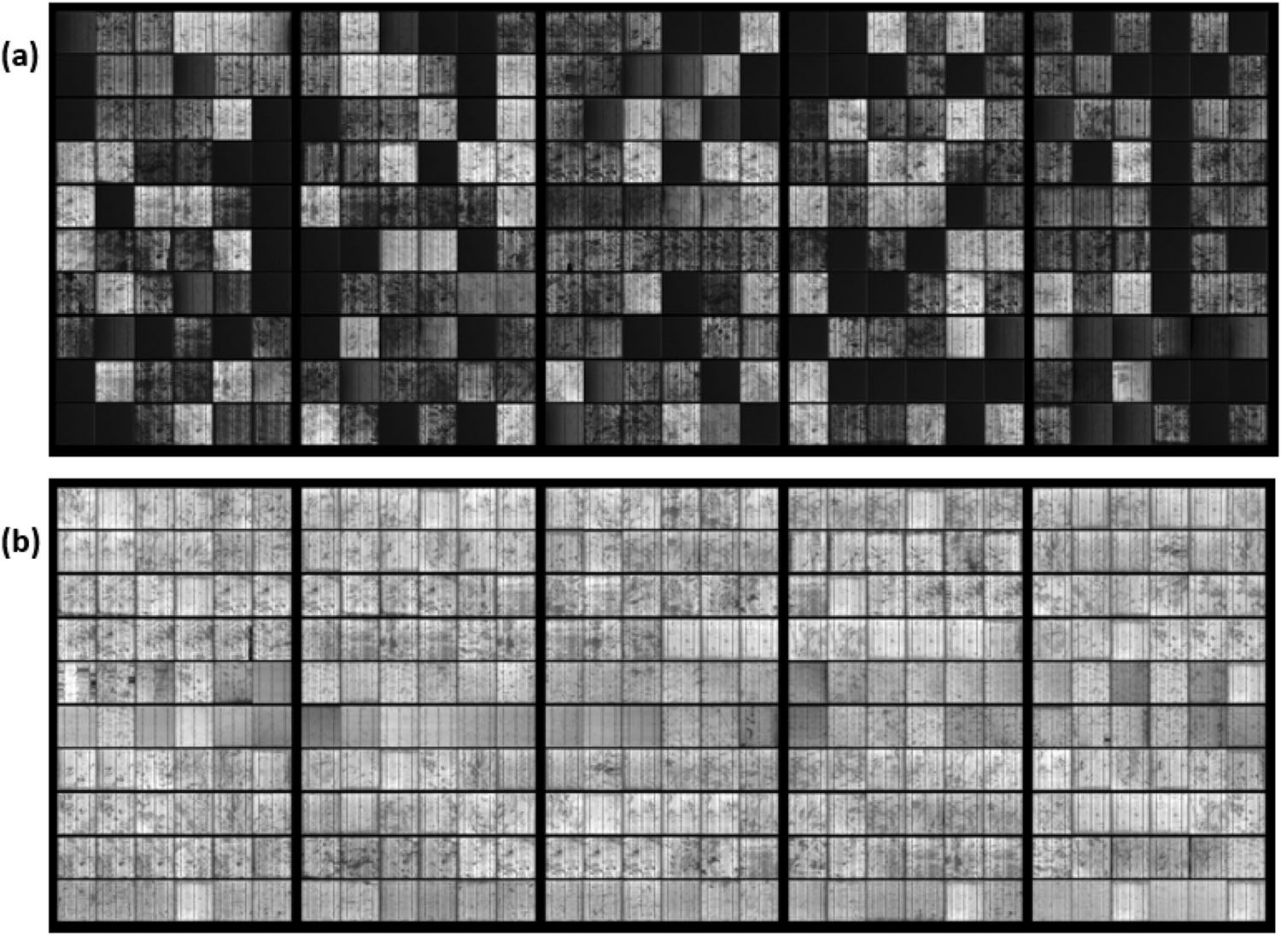
Electroluminescent image of two PV modules. (**a**) Modules located in a PID affected PV string (string 1). (**b**) Modules located in the healthy PV string (string 5). The electroluminescent images were taken during nigh-time, under an ambient temperature of 26 °C, and the modules were running at 90% of their short circuit condition.

The confirmed PID case in the PV strings was also supported by examining several PV modules under EL testing. For example, the output image of five PV modules from PV string 1 vs in the healthy PV string is shown in Fig. 3. Through EL detection, it is clear that the affected modules by PID, Fig. 3a, has low EL intensity compared with the healthy PV modules, Fig. 3b. In addition, the PID affected modules show shunting (blackout) cells that are likely to degrade the module's performance. However, unlike the healthy PV modules with homogenous EL intensity across the solar cells, these only have some micro cracks. Despite that some solar cells in the healthy PV modules have some cracks or minor shunting, these are typically normal to occur during the first year of operation and they have insignificant impact on the performance of the modules^22,23^.

**Figure 4:**
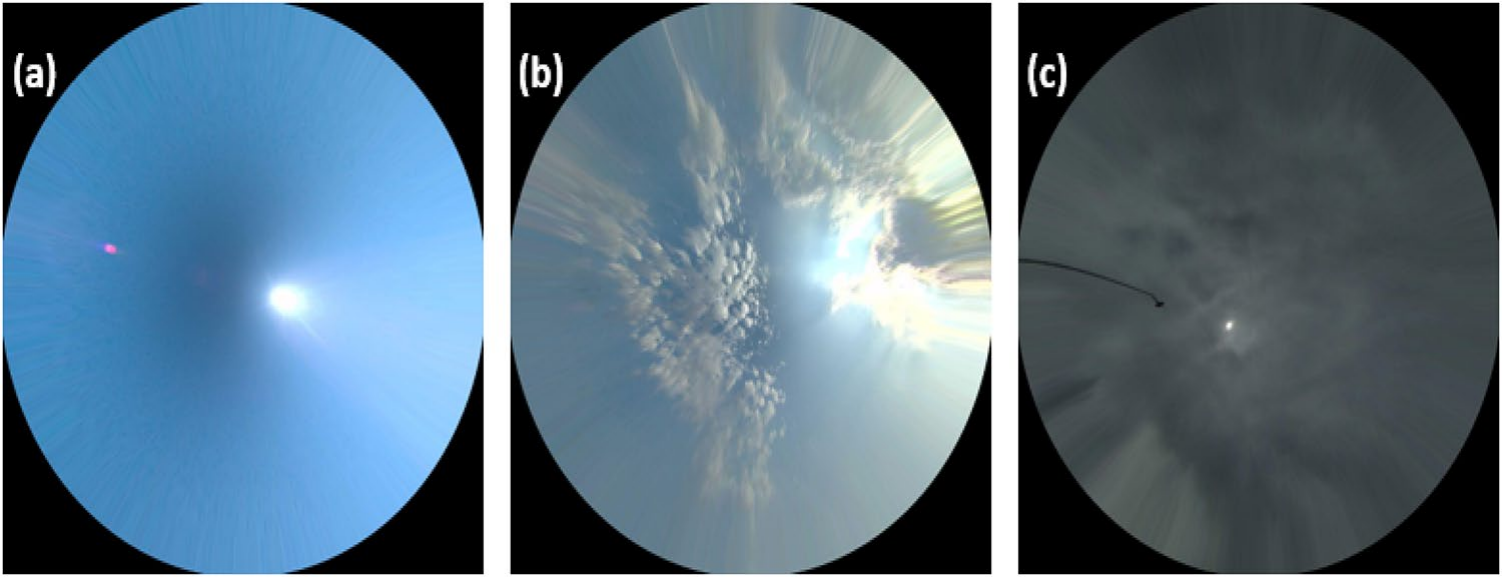
Sky image for the PV operation condition. (**a**) Clear sky. (**b**) Partial shading. (**c**) Overcasting. These images were taken using TSI 440A total sky imager. The temperature of the PV modules at these conditions is 26, 23 and 19 °C, respectively; and the solar radiation is 730, 677, and 408 W/m^2^.

A series connection of PV modules in Fig. 3 was used to test the I-V curve under a variety of field conditions. Figure 4a shows the modules under no shading (cloud-free skies), while Fig. 4b shows them under partial shading (some clouds). Last but not least, the modules are evaluated when operating under overcast conditions (Fig. 4c).

**Figure 5:**
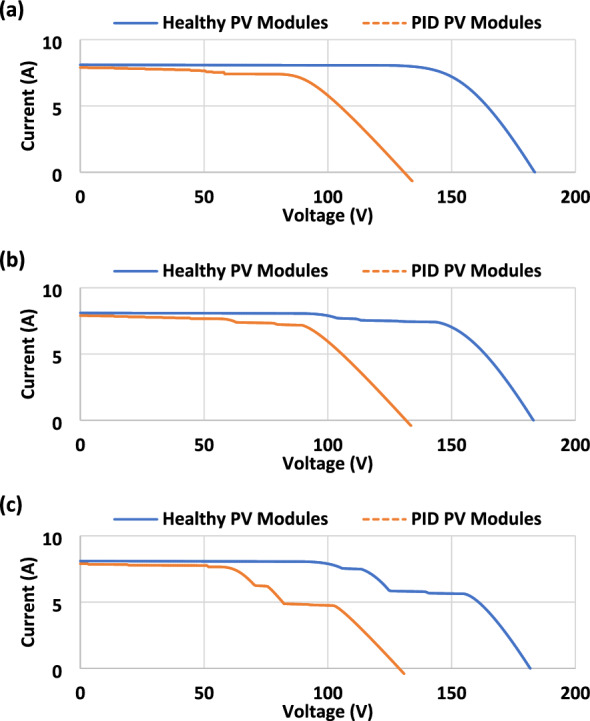
Current–Voltage (I–V) curve measurements of the examined PV modules. (**a**) Clear sky. (**b**) Partial shading. (**c**) Overcasting. The I-V curve tracking accuracy is ± 0.5%. The blue curves show the healthy PV modules (free PID), and the orange curves show the PID PV modules.

The I–V curves were taken for both the healthy and the PID PV modules during each different sky conditions (Fig. 5). In PID affected modules, I-V curves dropped steeply because PID has a significant impact on output voltage. According to Fig. 5a, the open-circuit voltage dropped from 183.6 V for the healthy modules to 134.1 V for the PID modules. PID-affected PV modules do not show a significant drop in short circuit current. In spite of this, the maximum current that can be extracted from the PID affected modules is less than that from healthy modules. As a result, the PID modules will generate less output power due to the voltage and current drop.

The PID causes leakage of the current in the PV modules, resulting in a decrease in the shunt resistance. The shunt resistance is necessary to keep at a high value because it determines the power output of the PV module, especially at low solar radiation conditions. Therefore, a typical reduction in the shunt resistance would accelerate the power reduction as in the case of PID affected modules.

Table 2 summarizes the electrical output parameters (compiled from Fig. 5) for the tested PV modules. Due to the reduced current and voltage, the PID modules had a significant drop in power at maximum power point (MPP). PID accelerates the power decrease (−39.69%) due to reduced shunting resistance at lower irradiance conditions (overcast, 408 W/m^2^). During clear sky and partial shading conditions, respectively, the output power of the PID-affected modules decreases by −37.15 and −36.29%. The I–V curve tracer endeavoured to track approximately the same current at I_MPP_ for the PID compared with the healthy modules at the clear sky and partial shading because the modules are relatively affected by high irradiance (> 650 W/m^2^). However, at low irradiance conditions (overcasting), there is a −8.54% loss in tracking the I_MPP_. On the contrary, it is incomprehensible to track the identical V_MPP_ due to the PID and the reduction in the shunt resistance.

**Table 2:** Electrical parameters of the tested PV modules.

PV Modules	Environment Condition: Clear Sky (26 °C, 730 W/m^2^)				
	V_OC_ (V)	I_SC_ (A)	V_MPP_ (V)	I_MPP_ (A)	P_MPP_ (W)
Healthy	183.6	8.1	143.9	7.66	1102
PID affected	134.1	8.0	90.3	7.67	692.6
Difference* (%)	−26.9	−1.23	−37.2	+ 0.13	−37.15

PV Modules	Environment Condition: Partial Shading (23 °C, 677 W/m^2^)				
	V_OC_ (V)	I_SC_ (A)	V_MPP_ (V)	I_MPP_ (A)	P_MPP_ (W)
Healthy	183.1	8.1	145.8	7.33	1069
PID affected	133.6	7.9	90.9	7.49	681.0
Difference* (%)	−27.0	−2.47	−37.6	+ 2.18	−36.29

PV Modules	Environment Condition: Overcasting (19 °C, 408 W/m^2^)				
	V_OC_ (V)	I_SC_ (A)	V_MPP_ (V)	I_MPP_ (A)	P_MPP_ (W)
Healthy	181.8	8.1	155.3	5.62	872.6
PID affected	130.8	7.9	102.4	5.14	526.2
Difference* (%)	−28.1	−2.47	−34.06	−8.54	−39.69

*The “difference” is calculated as the following: $\left[100-\left(\frac{PID electrical parameter}{Healthy electrical parameter}\times 100\right)\right]$. The negative sign explains how much loss in the relevant electrical parameter is induced due to the presence of PID.

These results explain the differences between healthy and PID modules under different environmental conditions. As a result, the power reduction of the PID affected modules is very high (> 36%), even under high irradiance conditions. Furthermore, under low-irradiance conditions, the output power of the PID-affected PV modules may be further reduced due to reduced shunting resistance.

### PID recovery

PV strings affected by PID were equipped with anti-PID boxes between the strings and the inverter. This anti-PID box reverses the voltage of the PV modules, and in effect, polarises all of the PV modules in order to recover the PID of −1000 V of the PV modules^24–26^. It was decided to fit the anti-PID box only on the affected PV strings on 06/08/2020 after three days of confirming the PID through the use of thermal images of the PV strings.

**Figure 6:**
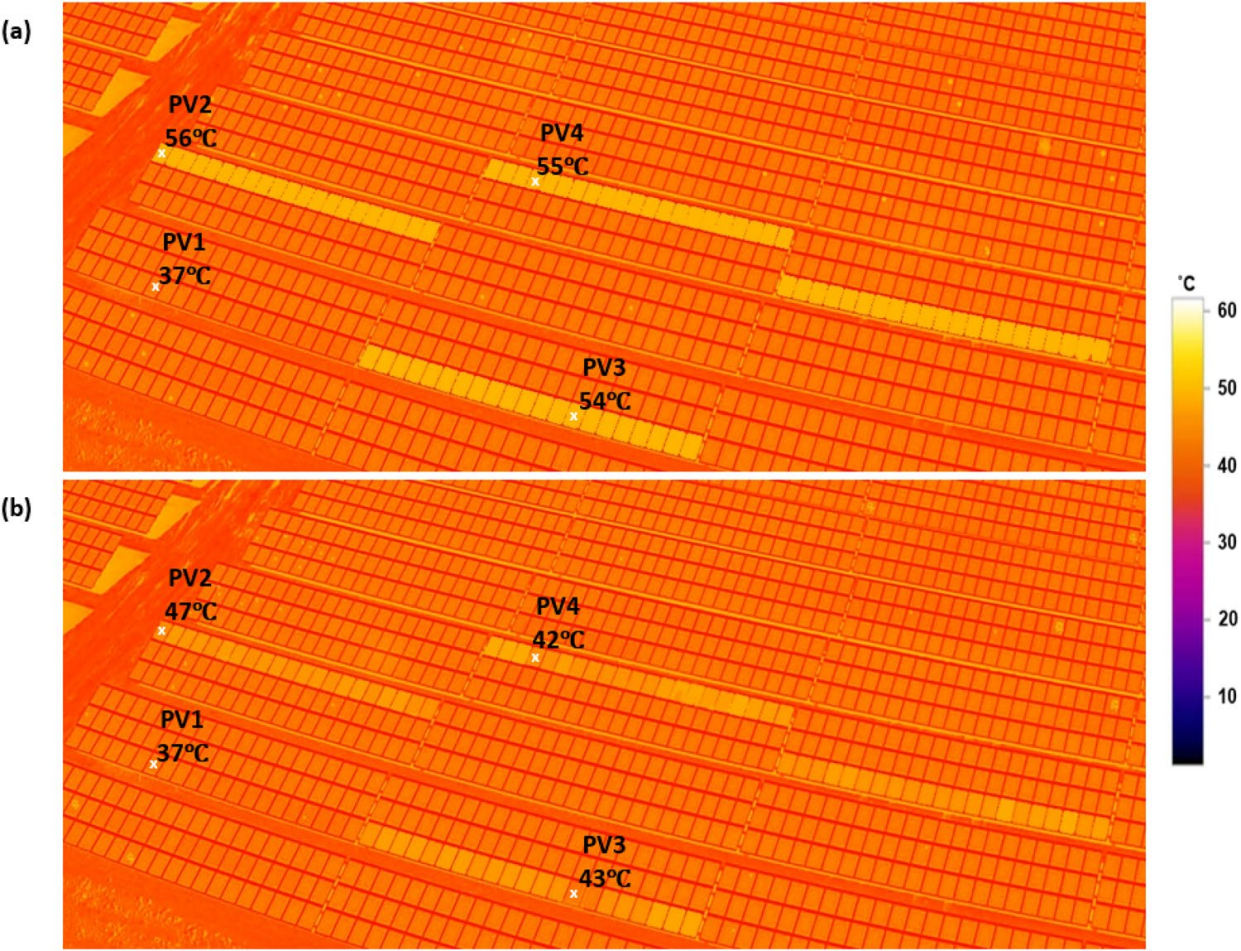
Thermography image of the tested PV strings during nigh-time. (**a**) Image taken on 03/08/2020, after one year of PV operation (ambient temperature 35 °C). (**b**) Image taken on 10/08/2021, after 1-year of fitting the anti-PID box on the PV strings (ambient temperature 35 °C). The thermal image is taken using DJI Mavic 2 Enterprise Advanced drone with thermal sensitivity ± 2 °C, the selected points on the figure are rounded to the nearest integer.

Figure 6 shows thermal images of PV strings after PID was confirmed (Fig. 6a) and after fitting the anti-PID box for a year (Fig. 6b). During the daytime, both images were taken under relatively the same ambient temperature.

As shown in Fig. 6b, the anti-PID box has partly improved the thermal performance of the PV modules. Three different instances were emphasised, where the PV surface temperature has dropped by nearly 9–13 °C; a summary of the results is presented in Table 3.

**Table 3:** Thermal difference between the selected PV modules.

Selected PV module	Surface temperature when the PID was confirmed (°C)	Surface temperature after 1-year of fitting the anti-PID box (°C)	Difference (℃)
PV1 “healthy PV module”	37	37	0
PV2	56	47	−9
PV3	54	43	−11
PV4	55	42	−13

**Figure 7:**
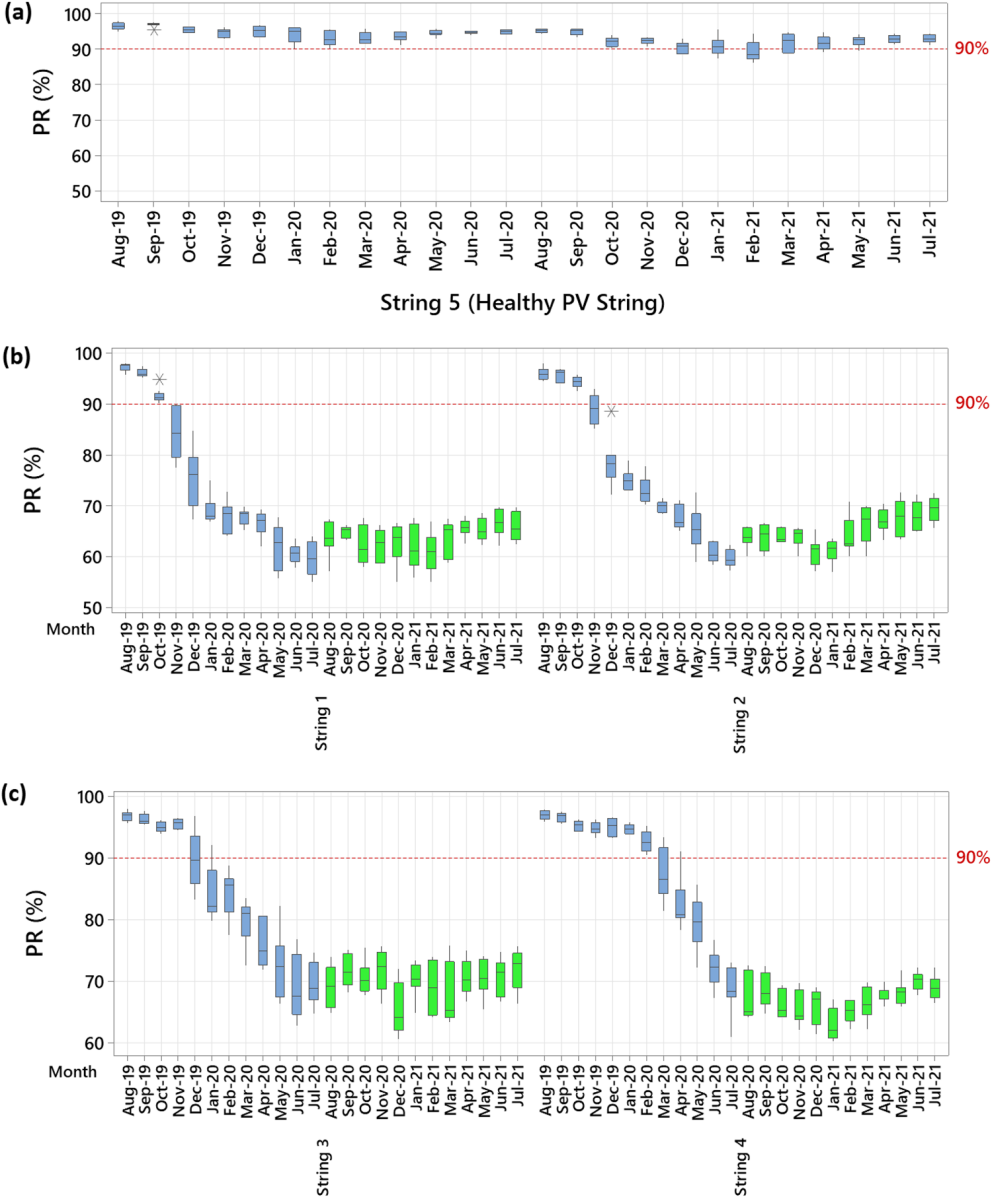
Performance ratio of the tested PV strings. (**a**) Healthy PV string (PV string 5). (**b**) PV strings 1 and 2. (**c**) PV string 3 and 4. The y-axis presents the PR ratio, and the baseline of 90% was selected to indicate low performance; the x-axis gives the month and the PV string number. This chart shows the PR ratio for the PV strings during their two years in the field, as well as the PR ratio after the anti-PID box is fitted.

In spite of the fact that the surface temperature of the modules decreased, the performance ratio of the PV strings did not change significantly because PID reduces the shunt resistance of PV modules permanently. In Fig. 7, you can see the monthly PR ratio of the PV strings for each month. As shown in Fig. 7a, the healthy PV module maintains a superior performance over a period of two years, with a PR ranging from 88 to 96%. PV strings (1, 2, 3 and 4) that were confirmed to have PID had exactly the same PR ratio, even when the anti-PID box was installed, their PR remained consistently below 75% even after the anti-PID box was installed, as shown in Fig. 7b,c.

The anti-PID recovery, however, stops the acceleration of power losses for the affected PID modules. Figure 7b,c demonstrate a virtually constant PR ratio after fitting the anti-PID box (see green highlighted boxplots), and no further reductions (i.e., below 50% PR ratio) have been observed. The results suggest that the anti-PID box has a positive impact on already PID-affected modules, which delays the recycling of the modules when a suitable detection mechanism is in place.

Figure 7 illustrates another conclusion about the long-term impact of PID on PV modules. The study shows that PID can severely affect modules, resulting in a 25% to 40% reduction in output power. Sadly, unidentified PIDs can cause rapid power losses, and we see this frequently in large industrial PV assets. Thus, our results highlight the importance of regularly inspecting PV systems to avoid system complications, particularly those related to PID.

**Figure 8:**
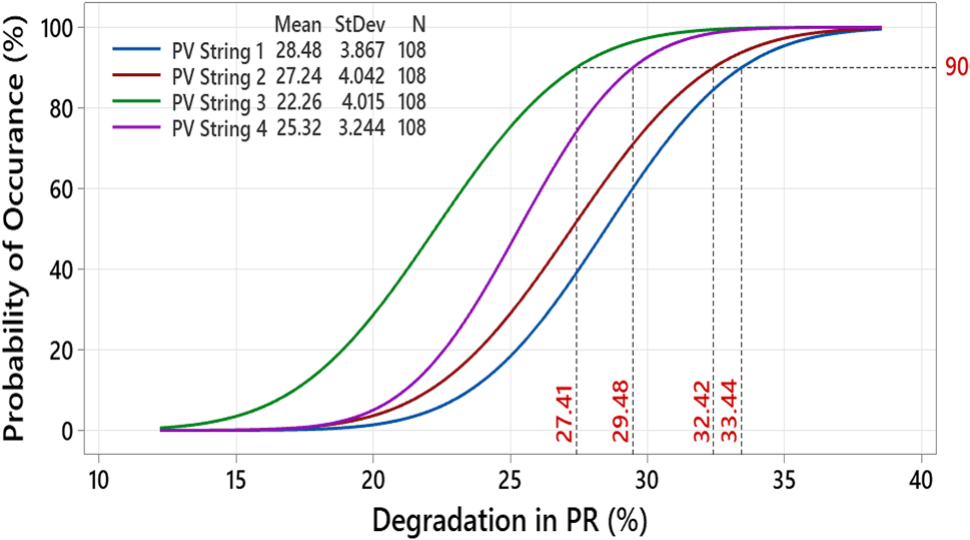
Plot of probability distribution functions for PV strings affected by PID. Probability of occurrence (in percentage) is represented by the y-axis, and degradation (in percentage) is represented by the x-axis. Plotting this figure requires data from Figs. 7b,c. N stands for the number of samples, and StDev for the standard deviation.

CDF is a widely used statistical analysis function that describes the probability of discovery of an investigated variable^27,28^. To explore the degradation in PR, we plotted the CDF for the affected PID PV strings as shown in Fig. 8. There is a 90% probability that a PV string affected by PID will have a PR degradation between 27.41 and 33.44%. Nevertheless, this assumption is only valid if the PID affected the modules for at least six months, as our previous data shows.

Also shown in Fig. 8 is the standard deviation (StDev) and the mean of the samples. According to the PR, the mean degradation ranges from 22.26 to 28.48%. A low StDev (3 to 4) indicates that the data are clustered around the mean. In summary, the CDF plot shows a degradation in PR for PV strings with affected PIDs. The probability of accelerated power losses in a PV string affected by at least 6-months of PID is approximately 27% to 33% with 90% confidence. Due to the location of our investigated PV system in Spain, Barcelona, these results may also be more accurate during hot summers and cold winters.

## Methods

### Photovoltaic system description

A polycrystalline silicon PV module with a peak output power of 220 W was investigated in this work. The modules have the following electrical characteristics:

- $$Isc = 8.18 A$$
- $$Voc = 36.6 V$$
- $$Impp = 7.67 A$$
- $$Vmpp = 28.7 V$$

A PV string consists of 21 series-connected PV modules, while a string box consists of four parallel-connected PV strings. The PV modules are single insulated. The PV Modules were installed in the field on a large-scale PV project with a capacity of 1.2 MW in northern Barcelona, Spain. The PV strings are connected to the ICONICA inverter for data monitoring and logging. Data is sampled at 1 sample/10 min. Local authorities have requested that the exact location of the PV system be kept confidential. It is relevant to note that all the pictures, data, and analysis presented in this paper have been approved by the partner company (Al-Marri Ltd).

Although our work only considers the impact of PID on polycrystalline silicon PV modules, some recent research has shown that PID can have the same severe impact on various PV technologies, such as p-type crystalline silicon passivated emitter and rear cell (PERC) solar cells^29^, monofacial PERC solar cells^11^, and bifacial PERC solar cells^30^.

### Thermal inspection

DJI Mavic 2 Enterprise Advanced drone was used to capture the thermal inspection of the PV system. The maximum tilt angle of this drone is 35 degrees, and it operates wirelessly. The thermal camera used is an uncooled VOx microbolometer, which has thermal sensitivity of 2%. A 35 mm lens and digital zoom up to 32 × are included with the camera.

### Electroluminescence inspection

A BrightSpot Automation EL-Camera Travel System was used to capture EL images of the PV modules. Digital camera kit with a removed infrared filter; the peak wavelength is 1150 nm, and the lens is 18–55 mm. PV modules were tested under 90% short circuit conditions to increase the EL output sensitivity (note, it can also be at 100% or a maximum of 110%). To prevent background light from deteriorating the quality of EL images, all EL tests were conducted at night (dark environment).

### Electrical parameters field measurements

PV200 PV tester was used to measure the current–voltage curves of the inspected PV modules. PV strings can be traced with up to 15 A short-circuit current and 1000 V open-circuit voltage with this instrument. The I–V curve tracking accuracy is + 0.5%, which means the voltage and current measurements can be within + 0.5%. The I–V were measured directly from the PV strings and downloaded via a USB connection to a personal computer.

### Sky imager

STI-440A total sky imager was used to check the cloudiness at PV site. Real-time images of the sky can be viewed via a web browser. Image resolution is 352 by 288 colour, 24-bit, and there is a rotating mirror with a shadow band. The sky imager is situated 30 m away from the PV system, and it can provide accurate cloudiness estimation/images for a 1 km radius.

## Anti-PID box

The anti-PID box, PROJOY Electric, was fitted between the affected PID PV strings and the inverter. This box can recover PID in the range 400–1000 V, and it has a load power of 3.75 W. It has 200 kΩ, minimum insulation resistance, and 3.3 mA maximum output current. The Anti-PID box prevents solar panels from losing power because of PID. The negative electrode of the DC string and the ground of the panels are connected in parallel with the inverter, so a high voltage (with a very low amperage) is generated. Therefore, the negative polarity between the negative electrode and ground allows the panel to be repaired at night by freeing it from the accumulated charge during the day.

### Performance ratio (PR) estimation

The performance ratio (PR) represents the performance of the PV system compared with the theoretical expected output power. For a typical PV system, a PR ratio above 90% stands very high. For example, in recent work on analysing 1000 polycrystalline silicon-based PV systems across the UK, it was found that the average PR is equal to 92.2%^31^. In this paper, the PR is calculated using Eq. (1) ^31^, where ${P}_{system}$ is the measured dc power of the PV strings in watts, ${P}_{rated}$ is the rated dc power of the PV string in watts, ${G}_{poa}$ is the plane-of-array solar radiation in W/m^2^, ${G}_{R}$ is the reference solar irradiation (1000 W/m^2^). The variable $\gamma$ presents the maximum power temperature coefficient of the PV modules at maximum output power (−0.33%/°C, taken from the PV modules’ datasheet), ${T}_{PV}$ is the PV modules surface temperature measured in °C, and ${T}_{R}$ is the PV modules surface reference temperature at standard test condition, 25 °C.

1 $$PR= \frac{{P}_{system}}{{P}_{rated} \frac{{G}_{poa}}{{G}_{R}} \left(1+ \gamma \left({T}_{PV}- {T}_{R}\right)\right)}$$

The relevant environmental parameters such as ${G}_{poa}$ and ${T}_{PV}$ are taken from a local weather station positioned near the PV installation. ${P}_{system}$ is taken from the data logger in the power converter unit connected with the PV strings.

In addition, in this paper, we have plotted the cumulative density function (CDF) for the PV strings affected by PID using Minitab software. The Degradation in the PR ratio is calculated using Eq. (2), where $PR ratio \left(\%\right)$ is the actual PR calculated using Eq. (1) for the PID affected PV strings, and ${PR ratio }_{Healthy}(\%)$ is the PR ratio calculated from the healthy PV strings (free PID string).

2 $$Degradation\, in\, PR=100-PR\, ratio \left(\%\right)-{PR\, ratio }_{Healthy}(\%)$$

In Eq. (2), the inclusion of the ${PR\, ratio }_{Healthy}(\%)$ is to subtract the actual losses of the PR in the healthy strings because this loss is anyways expected in PV modules. Therefore, this would result in an accurate estimation of the PR's exact reduction (degradation) due to the presence of PID in the affected PV strings.

## Data Availability

The dataset generated and analysed in this study may be available from the corresponding author (G.B.) on reasonable request.

## References

[1] Ma J (2018). Detection and assessment of partial shading scenarios on photovoltaic strings. IEEE Trans. Ind. Appl..

[2] Belhaouas N (2021). A new approach of PV system structure to enhance performance of PV generator under partial shading effect. J. Clean. Prod..

[3] Mansouri M, Trabelsi M, Nounou H, Nounou M (2021). Deep learning based fault diagnosis of photovoltaic systems: A comprehensive review and enhancement prospects. IEEE Access.

[4] Xiong Q (2018). Arc fault detection and localization in photovoltaic systems using feature distribution maps of parallel capacitor currents. IEEE J. Photovolt..

[5] Ahsan S, Niazi KAK, Khan HA, Yang Y (2018). Hotspots and performance evaluation of crystalline-silicon and thin-film photovoltaic modules. Microelectron. Reliab..

[6] Dhimish M (2020). Micro cracks distribution and power degradation of polycrystalline solar cells wafer: Observations constructed from the analysis of 4000 samples. Renew. Energy.

[7] López-Escalante MC (2016). Polyolefin as PID-resistant encapsulant material in PV modules. Sol. Energy Mater. Sol. Cells.

[8] Puranik VE, Gupta R (2021). Novel quantitative electroluminescence method for detailed performance analysis of PID-s affected crystalline silicon PV module. IEEE J. Photovolt..

[9] Sulas-Kern DB (2021). Electrochemical degradation modes in bifacial silicon photovoltaic modules. Prog. Photovolt..

[10] Dhimish M, Kettle J (2021). Impact of solar cell cracks caused during potential-induced degradation (PID) tests. IEEE Trans. Electron Dev..

[11] Carolus J (2019). Physics of potential-induced degradation in bifacial p-PERC solar cells. Sol. Energy Mater. Sol. Cells.

[12] Dhimish M, Tyrrell AM (2022). Power loss and hotspot analysis for photovoltaic modules affected by potential induced degradation. NPJ Mater. Degrad..

[13] Bedrich KG (2018). Quantitative electroluminescence imaging analysis for performance estimation of PID-influenced PV modules. IEEE J. Photovolt..

[14] Above. Thermographic and High-Definition Inspection (2022), https://www.abovesurveying.com/inspection/aerial-thermographic-solar-inspection/.

[15] Park HC, Lee SW, Jeong H (2020). Image-based gimbal control in a drone for centering photovoltaic modules in a thermal image. Appl. Sci..

[16] Hylský J (2018). Effect of negative potential on the extent of PID degradation in photovoltaic power plant in a real operation mode. Microelectron. Reliab..

[17] Bouaichi A (2022). Long-term experiment on p-type crystalline PV module with potential induced degradation: Impact on power performance and evaluation of recovery mode. Renew. Energy.

[18] Ma M (2021). Fault diagnosis of PID in crystalline silicon photovoltaic modules through IV curve. Microelectron. Reliab..

[19] Dhimish M, Hu Y, Schofield N, Vieira GR (2020). Mitigating potential-induced degradation (PID) using SiO2 ARC layer. Energies.

[20] Florides M, Makrides G, Georghiou GE (2020). Electrical and temperature behavior of the forward DC resistance with potential induced degradation of the shunting type in crystalline silicon photovoltaic cells and modules. IEEE J. Photovolt..

[21] Islam MA, Hasanuzzaman M, Abd Rahim N (2018). Investigation of the potential induced degradation of on-site aged polycrystalline PV modules operating in Malaysia. Measurement.

[22] Papargyri L (2020). Modelling and experimental investigations of microcracks in crystalline silicon photovoltaics: A review. Renew. Energy.

[23] Dhimish M, Lazaridis PI (2021). An empirical investigation on the correlation between solar cell cracks and hotspots. Sci. Rep..

[24] Hara K, Jonai S, Masuda A (2015). Potential-induced degradation in photovoltaic modules based on n-type single crystalline Si solar cells. Sol. Energy Mater. Sol. Cells.

[25] Dhimish M, Badran G (2022). Recovery of photovoltaic potential-induced degradation utilizing automatic indirect voltage source. IEEE Trans. Instrum. Meas..

[26] Sporleder K (2021). Quick test for reversible and irreversible PID of bifacial PERC solar cells. Sol. Energy Mater. Sol. Cells.

[27] Lanzante JR (2019). Evaluation and improvement of tail behaviour in the cumulative distribution function transform downscaling method. Int. J. Climatol..

[28] Jebeli M, Deiri E (2020). Estimation methods for the probability density function and the cumulative distribution function of the Pareto-Rayleigh distribution. Statistics.

[29] Li B (2021). Suppression of potential-induced degradation in monofacial perc solar cells with gradient-designed capping layer. Sol. Energy.

[30] Ma S (2022). Effective way to reduce rear-side potential-induced degradation of bifacial perc solar cells. Solar Energy Mater. Solar Cells.

[31] Dhimish M, Schofield N, Attya A (2020). Insights on the degradation and performance of 3000 photovoltaic installations of various technologies across the United Kingdom. IEEE Trans. Ind. Inform..

